# An Unusual “Collision Tumour” of the Prostate Gland and Rectum

**DOI:** 10.1155/2016/7486473

**Published:** 2016-04-20

**Authors:** C. F. Healy, D. Ferguson, M. F. Mohammed, J. Waterhouse, A. C. Harris

**Affiliations:** Department of Abdominal Imaging, Vancouver General Hospital, Vancouver, BC, Canada V57 1M9

## Abstract

Collision tumours of two different histopathological processes are rare. We describe a case of a patient with known low grade prostate adenocarcinoma developing a rectal GIST, which was diagnosed with combined imaging modalities of MR and ultrasound and confirmed by transrectal ultrasound guided biopsy.

## 1. Case Presentation

**Figure 1:**
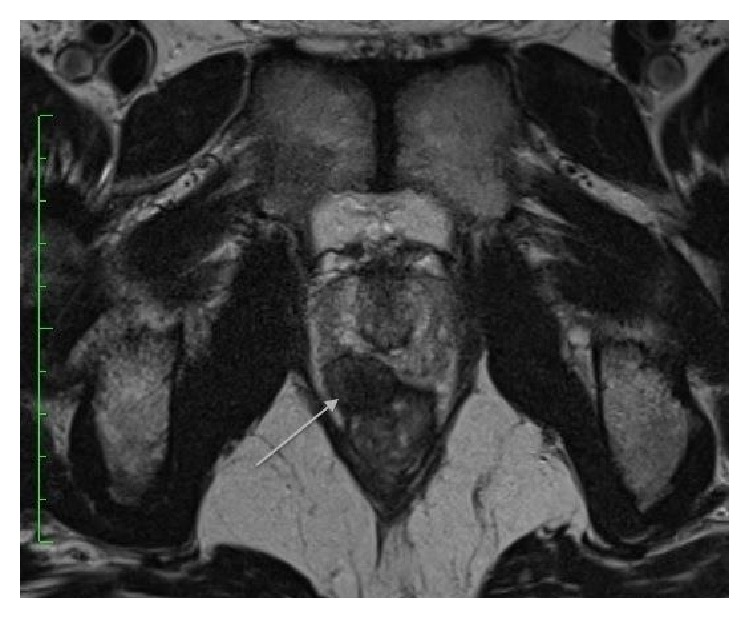
T2-weighted axial MRI at the level of the pubic symphysis demonstrating a T2 low signal intensity lesion adjacent to the right peripheral zone of the prostatic apex measuring 1.7 × 1.4 cm (arrow).

A 61-year-old Asian man with a history of low-risk prostate adenocarcinoma presented to his physician for an annual check-up. His prostate carcinoma was being treated conservatively with routine Prostate Specific Antigen (PSA) checks and annual digital rectal examinations (DRE). DRE revealed a smooth right-sided firm but mobile mass, which appeared within his prostate gland. At this time, his PSA was 1 ng/mL. The patient was referred for MRI of the prostate, which revealed a T2 low signal well-circumscribed nodule measuring 1.7 × 1.4 cm at the right apex abutting the peripheral zone of the prostate. The nodule appeared to be extracapsular and extraprostatic and appeared to involve the rectal submucosa and stretch the overlying rectal serosa and muscularis propria at 11 o'clock (Figure 1). The nodule was located approximately 6 cm cranial to the anal verge.

**Figure 2:**
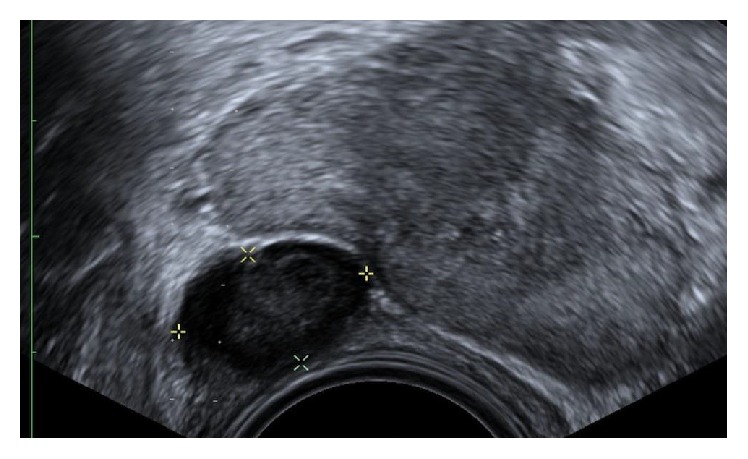
Transrectal ultrasound of the prostate gland. Image taken before core biopsy demonstrates a well-circumscribed lesion on the right, which is more convincingly extracapsular to the prostate gland and contains echogenic internal echoes (callipers).

The patient subsequently underwent a transrectal ultrasound guided prostate biopsy of this lesion. Ultrasound confirmed a well-circumscribed lesion at the right apex, which was more convincingly extraprostatic and rectal in origin (Figure 2). 18-gauge core biopsy of this lesion was performed, as well as 12 systematic core biopsies of the prostate gland.

**Figure 3:**
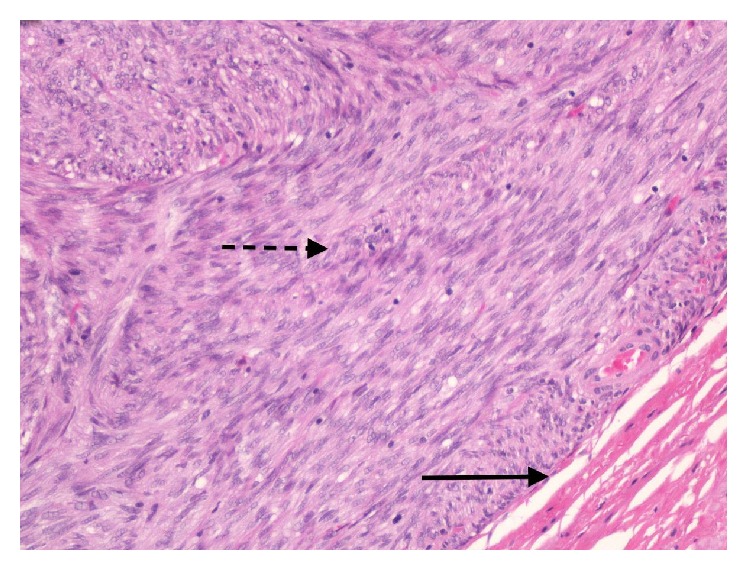
Low power view of the edge of the neoplasm demonstrating that it is sharply demarcated from normal rectal muscle (black arrow) and consists of interlacing fascicles of spindle cells with elongated nuclei (broken black arrow) (haematoxylin and eosin ×200).

Histological analysis of this nodule demonstrated almost complete replacement of the needle cores by a low-grade spindle cell mesenchymal neoplasm (Figure 3). No significant mitotic activity (<1/50 high-powered field) was identified. Spindle cells were positive for CD117 (c-kit) by immunohistochemistry. Findings were in keeping with a low-grade rectal gastrointestinal stromal tumour (GIST).

The prostate biopsies were positive for prostate adenocarcinoma, Gleason 3 + 3 (on 3 of 12 cores).

The patient underwent a successful R0 en bloc full thickness resection of the rectal tumour. The low-risk prostate cancer is being treated conservatively with active surveillance.

## 2. Discussion

Rectal GISTs are rare, accounting for 0.1% of all rectal tumours and approximately 5% of all GISTs [1, 2]. The most common symptoms are bleeding, a palpable mass, and rectal pain. GISTs are known to coexist with certain neoplasms, including pulmonary chondromas and paragangliomas (Carney's Triad). “Collision tumours” of two histologically distinct tumour types of the rectum and prostate gland are rare, with only a single case report published to date describing an anorectal GIST and prostate adenocarcinoma [3].

GISTs are best treated by surgery and are not radiosensitive or chemosensitive. The introduction of effective tyrosine kinase inhibition is considered the treatment of choice for patients with inoperable or metastatic disease. Controversy exists as to whether abdominoperineal resection (APR) or conservative surgery is the best alternative [4]. Due to its small size and lack of significant mitotic activity, local resection was the preferred treatment in the described case.

The detection of “collision tumours” of the prostate gland and rectum, although uncommon, is likely to increase with the increasing use of multiparametric MRI in the evaluation of prostate adenocarcinoma.
